# Facilitated/Pharmaco-invasive Approaches in STEMI

**DOI:** 10.2174/157340312803217157

**Published:** 2012-08

**Authors:** Davide Capodanno, George Dangas

**Affiliations:** 1 Ferrarotto Hospital, University of Catania, Catania, Italy; 2 Cardiovascular Institute, Mount Sinai Medical Center

**Keywords:** Percutaneous coronary intervention, myocardial infarction, facilitated, pharmacoinvasive.

## Abstract

Primary percutaneous coronary intervention (PCI) is the preferred reperfusion method in patients with ST-elevation myocardial infarction (STEMI), when performed in a timely manner and by skilled operators. However, this strategy has shown to be limited in environments with lack of PCI facilities and delay in the first medical contact-to-balloon time for logistic reasons. Pretreatment with fibrinolysis and/or glycoprotein IIb/IIIa inhibitors before PCI has the potential to provide early pharmacologic reperfusion before definitive PCI in STEMI patients. However, current data suggest that facilitated PCI does not offer any advantage over primary PCI. Conversely, a role for pharmacoinvasive recanalization, defined as pharmacological reperfusion followed by rapid transfer for routine delayed coronary angiography and PCI may still be considered in centers without on-site PCI capability.

## INTRODUCTION

Primary percutaneous coronary intervention (PCI) is the preferred treatment modality in patients with ST-Segment Elevation Myocardial Infarction (STEMI) referred to high volume, well-equipped hospitals with PCI capability [1,2], due to established superior rates of infarct-related artery patency and Thrombolysis In Myocardial Infarction (TIMI) 3 flow compared with thrombolytic therapy [3,4]. These positive effects on surrogate endpoints are proven to translate into decreased mortality, reverse ventricular remodeling and reduced cardiac dysfunction if primary PCI is performed in the early hours after the onset of STEMI [2,5,6]. In recent years there has been a notable interest and progress in the elaboration of transfer protocols for minimizing delays to primary PCI. On this background, the most recent US and European PCI guidelines set the first medical contact-to-balloon time goal to 120 minutes for interhospital transfer of STEMI patients, with emphasis on the need to strive for total ischemia times <90 minutes [1,2]. However, in a sizable proportion of patients, the effectiveness of STEMI reperfusion is still limited by delays in PCI [7]. In particular, there are environments where delays to primary PCI remain too long for logistic reasons, and alternative reperfusion strategies are needed [8]. The notion that STEMI patients in whom TIMI 3 flow is present before angioplasty present with greater clinical and angiographic evidence of myocardial salvage and have improved early and late survival [9] encouraged the design of prospective randomized trials of pharmacological strategies to promote early reperfusion before definitive mechanical intervention.

## FACILITATED PCI

**Fig. (1):**
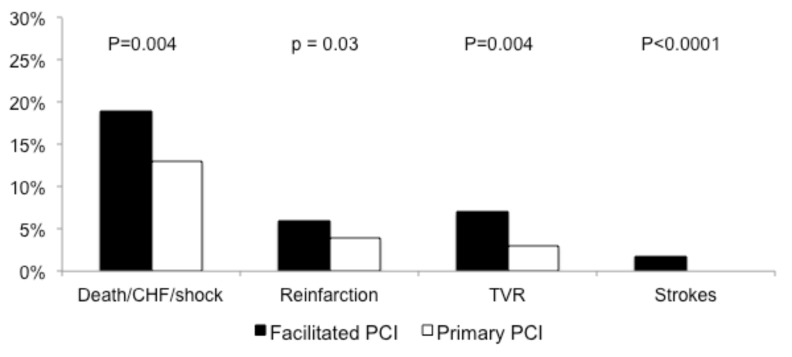
Ninety-day incidences of death, congestive heart failure (CHF) or stroke (primary endpoint), reinfarction, target vessel revascularization (TVR) and stroke in the Facilitated PCI (black) and Primary PCI (white) groups from the ASSENT 4 study. The trial was prematurely interrupted because of the observed increased risk of adverse events in the Facilitated PCI arm. PCI = percutaneous coronary intervention.

Using pharmacological agents (i.e., fibrinolytic drugs or half-dose fibrinolytic therapy plus glycoprotein IIb/IIIa inhibitors [GPI]) in patients with long delays to PCI has an intuitive appeal. This so-called “facilitated PCI” is expected to increase the window of opportunity in which primary PCI can be performed, by enabling early reperfusion followed by rapid transfer to a PCI facility. Despite being attractive, this concept has been frustrated by at least two large randomized trials. In the Assessment of Safety and Efficacy of a New Treatment Strategy for Acute Myocardial Infarction (ASSENT-4) trial, 1,667 STEMI patients were randomized to facilitated PCI with tenecteplase versus primary PCI alone [10]. The trial was prematurely interrupted because of the observed increased risk of adverse events in the facilitated arm, including death or heart failure (primary endpoint), intracranial hemorrhages and, paradoxically, ischemic events that were not attributed to bleeding (Fig. **1**). These latter surprising findings could be partly explained by the fact that clopidogrel treatment was suboptimal in either group and GPI were administered in only 10% of patients in the facilitated group. As a matter of fact, fibrinolysis is known to activate platelets and therefore adequate platelet inhibition is needed to avoid increased ischemic events. In addition, patients expected to benefit most from facilitated PCI (i.e., those presenting early after the onset of symptoms with long delays to PCI) were substantially under-represented in the trial.

**Fig. (2):**
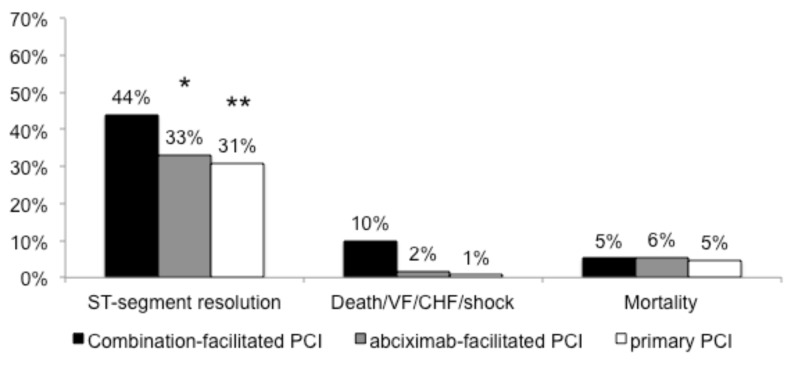
Ninety-day incidences of ST-segment resolution, the composite of death, ventricular fibrillation (VF) within 48 hours, congestive heart failure (CHF) or shock (primary endpoint), and death in the combined-facilitated PCI (black), abciximab-facilitated PCI (gray) and primary PCI (white) groups from the FINESSE study. Enrollment in the study was stopped at 82% of the originally planned sample size due to slow enrollment and financial overruns. There were no differences between treatment arms for the primary composite end point. PCI = percutaneous coronary intervention; * P=0.01 versus combined-facilitated PCI; ** P=0.003 versus combined facilitated PCI

The Facilitated Intervention With Enhanced Reperfusion Speed to Stop Events (FINESSE) trial, randomized 2,452 STEMI patients to facilitated PCI with abciximab, facilitated PCI with combination half-dose reteplase plus abciximab and primary PCI with abciximab given at the time of PCI [11]. Enrollment in the study was stopped at 82% of the originally planned sample size due to slow enrollment and financial overruns. There were no differences between treatment arms for the primary composite end point of all-cause mortality, readmission for heart failure, ventricular fibrillation, or cardiogenic shock or for any of the component endpoints (Fig. **2**). In addition, TIMI non-intracranial major bleeding and minor bleeding were significantly higher for the abciximab/lytic facilitated PCI strategy as compared with primary PCI. Differently from the ASSENT-4 trial, the FINESSE trial provided good platelet inhibition, but, once again, patients expected to benefit most from facilitated PCI were under-represented. On this background, a small trial recently sought to assess whether facilitated PCI after pre-hospital fibrinolysis with optimized concomitant antiplatelet therapy leads to smaller infarct size and better reperfusion and clinical outcomes in comparison with primary PCI in STEMI patients presenting early in a regional network with long transfer distances [12]. In line with the ASSENT-4 and FINESSE trials, there was again no benefit of facilitated PCI over primary PCI. In view of the above-mentioned data, it seems that the idea of a facilitated strategy in STEMI should be put aside and, in fact, this approach is not currently recommended by guidelines. However, there could be patient groups who might benefit from facilitated PCI who were not included or identified in these trials, such as subjects presenting very early (<90 minutes from chest pain onset), but this has to be established by specifically designed trials.

## PHARMACO-INVASIVE STRATEGIES

Similar to facilitated PCI, in the so-called pharmaco-invasive strategy fibrinolytic therapy is given at non-PCI hospitals to establish reperfusion and is followed by transfer to a PCI facility for urgent PCI. Differently from facilitated PCI, however, this approach has been validated in clinical trials versus fibrinolytic therapy rather than primary PCI. In the Combined Abciximab Re-teplase Stent Study in Acute Myocardial Infarction (CARESS-AMI), 600 STEMI patients were treated with a combination of half-dose reteplase and abciximab at a non-interventional center and randomly assigned to immediate transfer to the nearest interventional center for PCI or to management in the local hospital with transfer only in case of clinically indicated rescue PCI [13]. The primary outcome, a composite of death, reinfarction, or refractory ischemia at 30 days, was reduced in the immediate-PCI group. The rate of major bleeding showed a relative increase in the immediate group, but this was not significant because of the low absolute incidence of events.

**Fig. (3):**
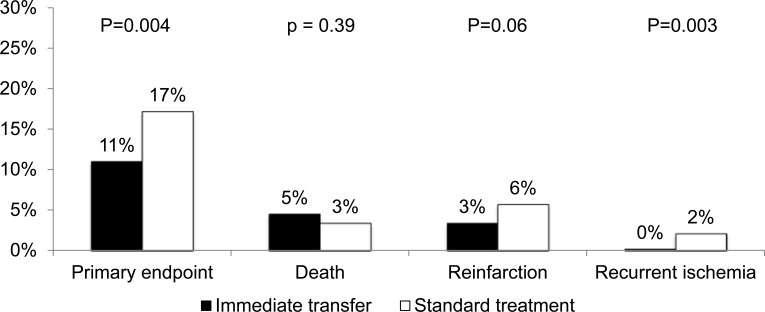
Thirty-day incidences of death, reinfarction, recurrent ischemia, new or worsening hear failure, or cardiogenic shock (primary endpoint), death, reinfarction and recurrent ischemia in the immediate transfer (black) or standard (white) groups from the TRANSFER-AMI study.

In the Trial of Routine Angioplasty and Stenting after Fibrinolysis to Enhance Reperfusion in Acute Myocardial Infarction (TRANSFER-AMI) trial, 1,059 STEMI patients receiving fibrinolytic therapy with tenecteplase at non-PCI centers were randomized either to a strategy of immediate transfer to another hospital and PCI (with the goal of performing coronary angiography and PCI of the infarct-related artery within 6 hours after fibrinolysis) or standard treatment, including clinically indicated rescue PCI [14]. Cardiac catheterization was performed in 98.5% of the patients in the immediate-transfer group at a median of 2.8 hours after randomization and 88.7% of the patients in the standard-treatment group at a median of 32.5 hours after randomization. Results showed a significant reduction in the primary composite end point of death, reinfarction, recurrent ischemia, new or worsening congestive heart failure, or cardiogenic shock within 30 days in the immediate-transfer group (Fig. **3**), with no significant differences in the rates of TIMI major or minor bleeding, transfusions or intracranial hemorrhages. These positive outcomes can be partly explained by the fact that, in the immediate-transfer group, PCI was performed early after fibrinolysis and only when persistent occlusion or substantial stenosis of the infarct-related artery was present. Together with progresses in procedural care leading to fewer bleedings (i.e., use of smaller sheaths, earlier removal of sheaths, radial access, the administration of lower doses of anticoagulants, and the elimination of postprocedural heparin infusions), the goal to perform catheterization and PCI in this optimal window after fibrinolysis may have allowed for better outcomes. Given the results of the CARESS-AMI and TRANSFER-AMI trials, the pharmacoinvasive strategy is currently endorsed by the US and European guidelines committees [1,2].

## CONCLUSIONS

The difference between facilitated PCI and pharmaco-invasive strategy is sometimes perceived as vague and elusive. What it is important to emphasize is that in the first case the decision to perform PCI is already taken before the additional pharmacological reperfusion treatment has been given, while in the second case PCI represents an invasive back-up implying transportation to a PCI hospital for either immediate rescue PCI in case of failed fibrinolysis or nonurgent coronary angiography to determine the need for additional revascularization of the culprit lesion.

Fibrinolytic therapy is the preferred reperfusion strategy at non-PCI centers unless transfer for primary PCI can be achieved with door-to-balloon times <90 to 120 min. Thrombolysis should be followed by a strategy of transferring patients to centers with PCI capabilities, although this pharmaco-invasive strategy (i.e., facilitated PCI) has not been shown to be beneficial compared with transfer for primary PCI alone, even with long delays to PCI. To decide which patients treated with fibrinolytic therapy should undergo coronary angiography (and when) is clinically challenging.

Unstable patients (e.g., those with severe heart failure or cardiogenic shock, hemodynamically compromising ventricular arrhythmias) not treated initially with primary PCI should undergo immediate coronary angiography with intent to perform PCI. Stable patients treated with fibrinolytic therapy and clinical suspicion of reperfusion failure (i.e., <50% ST-segment resolution 90 minutes after initiation of therapy) or infarct artery reocclusion should undergo immediate coronary angiography followed by PCI. In stable patients treated with fibrinolytic therapy and clinical evidence for successful reperfusion, coronary angiography should be performed between 3 and 24 hours. This time window is justified by the concern of thrombotic complications when an intervention is performed immediately following the administration of the lytic agent due to its prothrombotic effects and, on the other hand, of spontaneous re-infarction in the first day following thrombolysis.

Further studies are needed to establish if specific subgroups of patients (i.e., those who are fibrinolytic eligible, are at high clinical risk, present very early, and have very long delays to PCI) may still benefit from facilitated PCI compared with primary PCI [15]. In addition, current recommendations for facilitated PCI in STEMI deserve to be further tested on top of aspirin and bivalirudin or heparin against the availability of new P2Y12 receptor inhibitors with established greater effectiveness than clopidogrel, such as prasugrel and ticagrelor [16,17].
